# Vick's Floral Guide

**Published:** 1879-01

**Authors:** 


*Vick's Floral Guide.*—Of the many Guides and Seed and Plant Catalogues sent out by our Seedsmen and Nurserymen, and that are doing so much to inform the people and beautify and enrich our country, none are so beautiful, none so instructive as *Vick's Floral Guide.* Its paper is the choicest, its illustrations handsome, and given by the hundred, while its Colored Plate, representing in natural colors a group of paeonies, is a gem. This work, although costing but five cents, is handsome enough for a Gift Book, or a place on the parlor table. Published by James Vick, Rochester, N. Y.

The flower and vegetable seed of Mr. Vick, which the Editor of this JOURNAL has been using for a number of years are very superior, and cannot fail to give satisfaction to all who try them.

